# Conclusions reported in European Orthodontic Congress poster abstracts: are they based on clinical or statistical significance?

**DOI:** 10.1093/ejo/cjaf068

**Published:** 2025-10-22

**Authors:** Dawn Anne Xinying Yip, Martyn T Cobourne, Nikolaos Pandis, Jadbinder Seehra

**Affiliations:** Department of Orthodontics, Faculty of Dentistry, Oral & Craniofacial Sciences, King’s College London, Floor 21, Guy’s Hospital, Guy’s and St Thomas NHS Foundation Trust, Great Maze Pond, London SE1 9RT, United Kingdom; Department of Orthodontics, Faculty of Dentistry, Oral & Craniofacial Sciences, King’s College London, Floor 21, Guy’s Hospital, Guy’s and St Thomas NHS Foundation Trust, Great Maze Pond, London SE1 9RT, United Kingdom; Centre for Craniofacial Development & Regeneration, Faculty of Dentistry, Oral & Craniofacial Sciences, King’s College London, Floor 27, Guy’s Hospital, Guy’s and St Thomas NHS Foundation Trust, Great Maze Pond, London SE1 9RT, United Kingdom; Department of Orthodontics and Dentofacial Orthopedics, Dental School/Medical Faculty, University of Bern, Freiburgstrasse 7, Bern 3010, Switzerland; Department of Orthodontics, Faculty of Dentistry, Oral & Craniofacial Sciences, King’s College London, Floor 21, Guy’s Hospital, Guy’s and St Thomas NHS Foundation Trust, Great Maze Pond, London SE1 9RT, United Kingdom; Centre for Craniofacial Development & Regeneration, Faculty of Dentistry, Oral & Craniofacial Sciences, King’s College London, Floor 27, Guy’s Hospital, Guy’s and St Thomas NHS Foundation Trust, Great Maze Pond, London SE1 9RT, United Kingdom

**Keywords:** EOS Congress, orthodontic, abstracts, statistical significance, clinical significance, confidence intervals

## Abstract

**Background:**

*P*-values convey statistical significance while effect estimates and confidence intervals (CIs) place emphasis on the clinical significance. The aim of this study was to determine the frequency of reporting clinical or statistical significance in European Orthodontic Society (EOS) Congress scientific poster abstracts and to ascertain whether the conclusions drawn are based on either clinical or statistical significance.

**Materials and methods:**

Abstracts published between 2014 and 2024 were included. Pre-piloting and calibration were undertaken prior to data collection. Abstract characteristics were extracted independently by two reviewers. Descriptive statistics and frequency distributions were calculated.

**Results:**

A total of 3654 abstracts were analysed. The highest number of abstracts were presented in 2018 (13.0%). Epidemiological studies (cross-sectional, case-control, cohort, survey) (54.7%) were frequently presented, followed by laboratorial studies (17.4%) and systematic reviews (7.7%). No inferential statistics were commonly reported (58.5%). Within the abstracts reporting statistical significance (41.1%), typically only *P*-values were reported (32.5%), followed by the term ‘statistically significant’ stated only (3.7%), *P*-values in conjunction with estimates and 95% CIs (1.7%), *P*-values and 95% CIs (1.4%), 95% CIs (0.8%), estimates and 95% CIs (0.6%), and *P*-values and estimates (0.4%). When interpreting the reported results in the conclusion section, these were typically based on *P*-values (31.2%) or the term ‘statistically significant’ stated only, without consideration of outcomes between groups (14.7%). 95% CIs (0.7%), *P*-values and 95% CIs (1.3%), *P*-values in conjunction with estimates and 95% CIs (1.6%) and estimates and 95% CIs (0.5%) were infrequently considered. Across the study timeframe (per year), the results and author conclusions tended to be based on *P*-values primarily.

**Limitations:**

Only one society congress was assessed which may impact the generalizability of the results.

**Conclusions:**

Clinical significance is often under-reported in abstracts presented at EOS. Where applicable the reporting of clinical significance (effect size with CIs) and their interpretation in poster abstracts should be stipulated as this allows clinicians to gauge both the size and range of the observed differences between groups and the relevance to their clinical practice.

## Introduction

Annual scientific conferences facilitate the gathering of clinicians and academic professionals and serve as a valuable route for the sharing of new knowledge and research findings [1, 2]. In these forums the dissemination of scientific information is commonly undertaken in the form of abstract presentations (oral or poster) [3, 4]. Abstracts are concise summaries of research findings that allow the distribution of novel information to large groups of attendee’s research as well as to provide a baseline to direct future research efforts [5, 6].

The advantages of abstract presentations are plentiful; however, these should be tempered against their limitations. Word count restrictions can hinder the amount of information authors are able to provide, which can be insufficient to ascertain the scientific rigour of the study [7]. Usually only conference delegates are privy to abstracts, which restricts accessibility [8]. The latter is also compounded by the fact that the full-text publication rate of studies presented as abstracts at conferences has been found to range between 37.3% and 52.2% [3, 6, 9, 10]. Furthermore, abstracts may often present initial conclusions based on incomplete/preliminary results [11]. Subsequently, the findings and direction of the primary outcome may differ in the future respective full-text publications [3, 12, 13]. This could negatively impact patient care as clinicians who attend these scientific meetings may use findings to influence their clinical practice [4].

Statistical analyses such as inferential statistics are based on making observations in a sample of subjects and extrapolating these to make inferences about the larger population [14]. The two hypotheses tested are the null hypothesis (H_0_), which is the proposal that no change or difference exists between groups, and the alternative hypothesis (H_1_ or H_a_), which is the statement that there is an observed effect or difference between groups. The *P*-value, commonly selected as a value of 0.05 (5%) is the probability of finding the observed treatment effect or a more extreme one (i.e. rejecting the null hypothesis/*P*-value of less than 0.05) by chance alone given that the null hypothesis is true. *P*-values have remained the centre of numerous discussions and debates through the years [15]. They have value as an objective, standardized metric that allows comparison and bridging of outcomes across studies aiming to answer the same scientific question [16]. They also support evidence on a continuous rather than binary scale. The smaller the *P*-value, the stronger the evidence for rejecting the null hypothesis; hence, it provides a justification of the study findings as opposed to simply accepting or rejecting the null hypothesis [15].

However, the widespread use of *P*-values is accompanied by pervasive misinterpretation and consequently inappropriate application in medical research. One common misconception surrounding the *P*-value is that it reflects the probability of the null hypothesis being true [16, 17]. Based on this understanding, studies that do not find a statistically significant difference between groups assume there is no difference between the groups, whereas the true meaning of a lack of statistical significance is merely that there is insufficient evidence of a difference between groups [18, 19]. Since the definition of the *P*-value is the probability of finding a difference as extreme or more extreme than what is observed by chance alone given that there was no actual difference, for a *P*-value of .05, calculations have shown that the probability that there is no difference in treatment effect is not 5% but instead at least 28.9% [20]. Additionally, the *P*-value is deduced based on the premise that the null hypothesis is true. Thus, if the null hypothesis is rejected, the *P*-value is invalidated [17]. Another shortfall of the *P*-value is its data-dependent nature and sensitive to sample sizes and standard deviations [21, 22]. As the sample size increases and the standard deviation decreases, the *P*-value becomes smaller. As such, a large sample size can yield a significant *P*-value even with a very small effect size. Such results offer little clinical insight and are often not reproducible [15]. Lastly, although the *P*-value offers the opportunity to gauge strength of evidence based on its size, the common use of the 5% significance level has led to a binary division of research findings as either significant or non-significant [19]. It is important to note that this 5% threshold is an arbitrary value based solely on convention and should not form the basis for clinical decisions [22]. While the *P*-value undoubtedly has its place in medical research, its use in isolation to ascertain the effectiveness of treatment interventions is debated [21].

The more clinically appropriate approach would be to present together with the *P*-value the actual effect size and its range through the reporting of confidence intervals (CIs) [23, 24]. A 95% CI is the range within which we can assume, with 95% confidence, the true population mean lies [14]. It is presented in the original unit of measurement, thus offering a more clinically relevant interpretation by showing the range of the possible effects or association between groups to help determine if these observed differences suggest a true advantage of one treatment over the other [24]. CIs allow a shift in interpretation of results from the qualitative insight provided by *P*-values to a quantitative estimate of the effect [25]. The reporting of CIs is of even more importance when a non-significant difference is found as a judgement can be made on the clinical significance of the finding despite the absence of statistical significance [23, 24]. They also serve as an indication of the precision of the sample study estimates compared with the true population values [26]. If the sample size is small, the range of the CI will be wider, thus reflecting the amount of random error in the sample [14]. While an increase in sample size may artificially lower the *P*-value, its effect on the CI is to narrow its range around the same size of effect [23]. However, there are two points to be noted when using CIs in data analysis. Firstly, the reporting of CIs is not appropriate in all situations, such as when the data is descriptive in nature or when their use will not provide any clinical insight to the research findings. Secondly, while CIs help to show the effect of sampling variation on the precision of the study estimates, they cannot control for discrepancies that arise outside of sampling errors such as biases in study design or analysis [26]. In summary, *P*-values convey statistical significance while CIs place emphasis on the clinical significance of findings [27].

The over-emphasis on *P*-values and under-utilization of CIs have long been recognized in the field of biomedical research [24, 28–35]. However, the reporting frequency of clinical and statistical significance amongst conference abstracts is largely unknown. For many clinicians who attend conferences, the conference abstracts are often their only opportunity to determine if the reported findings are relevant to their clinical practice. Therefore, the aim of this present study was to determine the frequency of reporting clinical or statistical significance in European Orthodontic Society (EOS) Congress scientific poster abstracts and to assess whether the reported conclusions drawn are based on either clinical or statistical significance.

## Materials and methods

### Eligibility criteria

All European Orthodontic Congress scientific poster abstracts published between 2014 and 2024 were included. The 2020 scientific abstracts were excluded from this investigation as these were not published as the Congress was not held in person due to the COVID pandemic. Abstracts for oral presentations were excluded.

### Screening and selection of relevant abstracts

The abstract book of each Congress was reviewed independently by two reviewers (D.Y. and J.S.). Each abstract was assessed independently by two reviewers (D.Y. and J.S.). Disagreements were discussed between both reviewers until a consensus was reached (100% agreement was achieved).

### Data extraction

Data from each eligible abstract were extracted by two reviewers (D.Y. and J.S.) independently. Any disagreements were discussed between the reviewers and discrepancies resolved by discussion with the reviewer who has extensive experience in the conduct, methodological design, analysis, and reporting of studies (N.P.). A standardized and pre-piloted data extraction form was used. Prior to data extraction, the reviewers (D.Y. and J.S.) individually undertook an initial pilot calibration based on ten abstracts. The results were discussed between the reviewers (D.Y. and J.S.). Any disagreements were resolved by discussion with a third reviewer (N.P.). 100% agreement was achieved.

At the abstract level the following characteristics were extracted: year of publication, number of authors, type of study (randomized clinical trial (RCT), non-randomized prospective, epidemiological study (cross-sectional, case-control, cohort, survey), laboratorial study, systematic reviews (qualitative and quantitative), case report or series, narrative review, product review or clinical technique and other including audit and guidelines), centre (single or multi), statistical significance of primary outcome (significant, non-significant, non-applicable, or unclear), reporting of results (*P*-values only, 95% CIs only, estimates only, *P*-value and 95% CIs, estimate, *P*-value and 95% CIs, estimates and 95% CIs, the term ‘statistically significant’ stated only and no inferential statistics reported), and interpretation of results in conclusions (based on the following: *P*-values only, 95% CIs only, estimates only, *P*-value and 95% CIs, estimate and 95% CIs, estimate, *P*-value and 95% CIs, the term ‘statistically significant’ only stated and the results of inferential statistics were not considered). To ensure consistency in the interpretation of variables during data extraction the authors referred to the following articles [36–42]. The protocol for this study was not registered.

### Statistical analysis

Descriptive statistics and frequency distributions for each abstract characteristic were calculated. Trends in the reporting of results and interpretation of results reported in conclusions section of abstracts (per year) are visually presented. All analyses were conducted using the Stata 18 (Stata Corp, TX, USA).

## Results

A total of 3654 (*n* = 3654) abstracts were analysed. The highest number of abstracts was presented in 2018 (*n* = 476, 13.0%) with the lowest number in 2021 (*n* = 227, 6.2%). Typically, in the abstracts, five authors (*n* = 1387, 38.0%) were cited, who were more likely to be from the same institution (*n* = 2296, 62.8%). In the study timeframe, epidemiological studies (cross-sectional, case-control, cohort, survey) (*n* = 2000, 54.7%), followed by laboratorial studies (*n* = 635, 17.4%), systematic reviews (*n* = 283, 7.7%), other (audit, guidelines, etc.) (*n* = 243, 6.7%), and RCTs (*n* = 162, 4.4%) were frequently presented. The primary outcome was more likely to be reported as statistically significant (*n* = 1503, 41.1%) (Table 1). Per year of EOS Congress there was a preponderance for presenting epidemiological studies and laboratorial studies compared with other study types (Supplementary Figure S1).

**Table 1.** Characteristics of abstracts presented at EOS Congress.

Year of congress	*N* (%)
2014	419 (11.5)
2015	379 (10.4)
2016	408 (11.2)
2017	434 (11.9)
2018	476 (13.0)
2019	365 (10.0)
2021	227 (6.2)
2022	229 (6.3)
2023	358 (9.8)
2024	359 (9.8)

Number of authors	*N* (%)
1	150 (4.1)
2	471 (12.9)
3	787 (21.5)
4	838 (22.9)
5	1387 (38.0)
6	18 (0.5)
7	3 (0.1)

Centre	*N* (%)
Single	2296 (62.8)
Multi	1358 (37.2)

Study type	*N* (%)
Randomized clinical trial	162 (4.4)
Non-randomized prospective	112 (3.1)
Epidemiological study	2000 (54.7)
Laboratorial study	635 (17.4)
Systematic review	283 (7.7)
Case report or series	71 (1.9)
Narrative review	87 (2.4)
Product review or clinical technique	61 (1.7)
Other (audit, guidelines, etc.)	243 (6.7)

Statistical significance primary outcome	*N* (%)
Non- significant	409 (11.2)
Significant	1503 (41.1)
^a^Not applicable or unclear	1742 (47.7)
Total	3654 (100)

^a^study type was case report or series, narrative reviews, product review or clinical technique and other.

**Figure 1.**
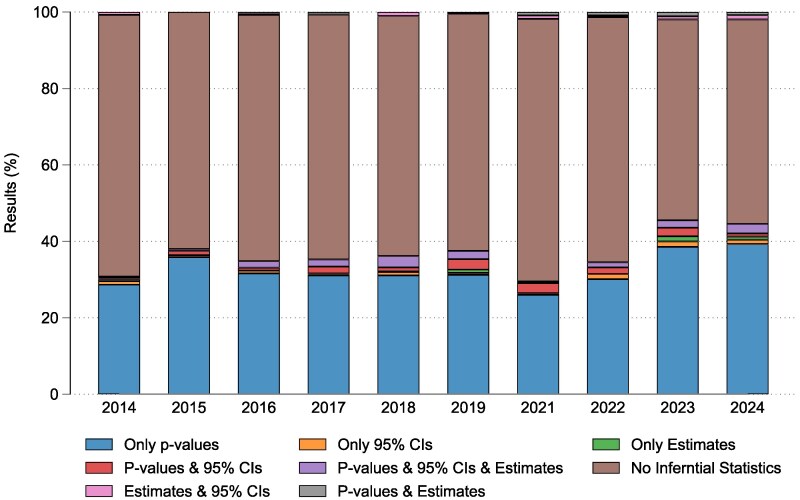
The distribution of the statistical results reporting in abstracts per congress year.

Regarding the statistical analysis reported in the results section of abstracts, no inferential statistics were commonly documented (*n* = 2136, 58.5%). Within the cohort of abstracts reporting statistical significance (*n* = 1503, 41.1%), typically *P*-values were reported (*n* = 1189, 32.5%) followed by the term ‘statistically significant’ stated only (*n* = 135; 3.7%), *P*-values in conjunction with an estimate and 95% CIs (*n* = 61, 1.7%), *P*-values and 95% CIs (*n* = 52, 1.4%), 95% CIs (*n* = 29, 0.8%), estimates and 95% CIs (*n* = 21, 0.6%), and *P*-values and estimates (*n* = 16, 9.4%) (Table 2). The trend of frequently reporting *P*-values was consistent across the study timeframe with approximately a third of abstracts per year citing *P*-values (range: 26.0%–39.3%) (Fig. 1) (Supplementary Table S1). Per congress year statistically significant results were more likely to be reported than clinically relevant results (Supplementary Figure S2).

**Table 2.** Reporting of the statistical results in abstracts across all time periods.

Results	*N* (%)
Only *P*-values	1189 (32.5)
Only 95% CIs	29 (0.8)
Only estimates	15 (0.4)
*P*-values and 95% CIs	52 (1.4)
*P*-values, 95% CIs and estimates	61 (1.7)
Estimates and 95% CIs	21 (0.6)
*P*-values and estimates	16 (0.4)
The term ‘statistically significant’ stated only	135 (3.7)
No inferential statistics reported	2136 (58.5)
Total	3654 (100.0)

**Figure 2.**
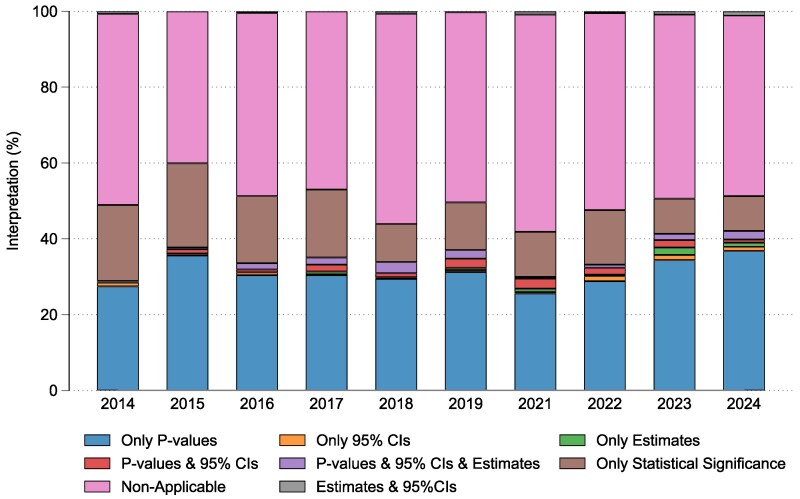
The distribution of the conclusions based on the interpretation of the type of statistical results (per congress year).

When interpreting the reported results in the conclusion section of abstracts, in just under half the sample the results of inferential statistics were not considered (*n* = 1805, 49.4%). When authors had interpreted the results, the conclusions were typically based on *P*-values (*n* = 1139, 31.2%) or the term ‘statistically significant’ stated only, without consideration of outcomes between groups (*n* = 538, 14.7%). 95% CIs (*n* = 27, 0.7%), *P*-values and 95% CIs (*n* = 49, 1.3%), *P*-values in conjunction with an estimate and 95% CIs (*n* = 57, 1.6%), and estimates and 95% CIs (*n* = 19, 0.5%) were infrequently considered (Table 3). Similar to the results reporting, across the study timeframe (per year), the author conclusions tended to be based on considering *P*-values primarily (Range 25.6%-36.8%) (Fig. 2) (Supplementary Table S2). Per congress year the conclusions of abstracts were based on the interpretation of statistically significant results rather than clinically relevant results (Supplementary Figure S3).

**Table 3.** Interpretation of statistical results reported in conclusions section of abstracts across all time periods.

Interpretation and conclusions	*N* (%)
Based on *P*-values only	1139 (31.2)
Based on 95% CIs only	27 (0.7)
Based on estimates only	20 (0.6)
Based on *P*-values and 95% CIs	49 (1.3)
Based on *P*-values, 95% CIs and estimates	57 (1.6)
Based on estimates and 95%CIs	19 (0.5)
The term ‘statistically significant’ stated only, without consideration of outcomes between groups	538 (14.7)
The results of inferential statistics were not considered	1805 (49.4)
Total	3654 (100)

At the level of each study type, the interpretation of results reported in the conclusions section was typically based on *P*-values. The frequency of interpreting the results based on *P*-values and 95% CIs and estimates in the conclusions for each study type was RCT (1.9%) non-randomized prospective (2.7%), epidemiological study (1.1%), laboratorial study (0.2%), systematic reviews (9.2%), and narrative review (3.4%) (Supplementary Table S3 and Supplementary Figure S4).

## Discussion

This review found a persistent over-reliance on *P*-values and under-reporting of CIs amongst scientific poster abstracts presented at EOS Congress over the past 10 years. Within the abstracts that reported inferential statistics, 32.5% reported *P*-values in the results section of the abstract. Furthermore, when authors had interpreted the results, the conclusions were typically based on *P*-values (31.2%) or the term ‘statistically significant’ stated only, without consideration of outcomes between groups (14.7%) (Illustrations of poster abstracts are shown in Supplementary File S1). Throughout the study time frame (10-years), the proportion of studies that both reported *P*-values and based their conclusions primarily on *P*-values remained relatively constant with no indication of improvement over time.

The overall frequency of CI reporting amongst the results section of abstracts that performed inferential statistics was low at 10.7% (163/1518). There is a paucity of studies investigating the reporting of clinical significance in conference abstracts; however, comparisons can be drawn from studies investigating CI reporting in article abstracts and full text. The reporting of CIs amongst studies published in three orthodontic journals was between 4% and 8% [24]. Similarly, in a review of nine leading prosthodontic and dental implantology journals, 14% of published studies reported CIs [30]. The situation is similar in article abstracts. In an assessment of abstracts published between 2010 and 2015 in 15 leading psychiatric journals, 22% of abstracts reported CIs [29]. In a quantitative review of statistical reporting trends in the abstracts of RCTs indexed on PubMed the reporting of CIs increased from 0.5% in 1975 to 29% in 2021 [34]. Compared with these findings our results show a considerably lower proportion of abstracts reporting CIs.

An over-reliance on significance testing within abstracts was observed with almost 1 in 3 (32.5%) reporting *P*-values in isolation without estimates or CIs. This finding was comparable to a large scale investigation on *P*-value reporting in 29 725 abstracts of articles from 151 core clinical journals in 2014 which found a *P*-value reporting rate of 33.0% [28]. A systematic analysis of published abstracts in five major medical journals and seven major epidemiological journals highlighted that the reporting of CIs in abstracts improved by 75% from 1970 to 2014 [32]. Unfortunately, unlike the speciality of orthodontics, the published literature in other fields appears to be showing an increasing awareness of the value of CI reporting. Lastly, when interpreting the findings of inferential statistics in the conclusions of abstracts almost 46% authors based their conclusions on *P*-values alone (31.2%) or labelled their findings as ‘statistically significant’ (14.7%) without elaborating on the clinical importance of their findings. This should not be unsurprising as there is a bias towards publishing studies with statistically significant effect estimates within dental literature [43].

The observed reliance on *P*-values and statistical significance has been a perennial challenge in the biomedical literature. To a researcher/clinician, *P*-values may appear to be a deceptively simple way to analyse and present the findings of their study. Since the introduction of *P*-values in 1900 [44], *P*-values have been artificially simplified over time into the dichotomy of significance and non-significance, which may then be used to superficially validate study results. This important point is highlighted in a statement made by the American Statistical Association which stated that the ‘widespread use of “statistical significance” (generally interpreted as “*P* ≤ 0.05”) as a licence for making a claim of a scientific finding (or implied truth) leads to considerable distortion of the scientific process’ [45]. The statements six principles are summarized in Table 4.

**Table 4.** Six principles which address misconceptions and misuse of the *P*-value.

Principles of misconceptions and misuse of *P*-values
*P*-values can indicate how incompatible the data are with a specified statistical model.
*P*-values do not measure the probability that the studied hypothesis is true, or the probability that the data were produced by random chance alone.
Scientific conclusions and business or policy decisions should not be based only on whether a *P*-value passes a specific threshold.
Proper inference requires full reporting and transparency.
A *P*-value, or statistical significance, does not measure the size of an effect or the importance of a result.
By itself, a *P*-value does not provide a good measure of evidence regarding a model or hypothesis.

In contrast, the reporting of an estimate that includes CIs is considered the most informative reporting of statistical inference [25]. So how can the latter be encouraged? One such initiative is to actively implement the use of reporting guidelines. Currently, submission of EOS Congress poster abstracts does not require adherence to any reporting guidelines. CIs were more likely to be reported in RCTs, possibly due to the implementation of CONSORT guidelines which recommend the reporting of estimated effect sizes and its precision [30]. However, the current investigation has highlighted the general lack of awareness of reporting and interpreting 95% CIs across all study types including RCTs with *P*-values typically favoured (range 54.3%–2.1%) (Supplementary Table S3 and Supplementary Figure S4).

CI reporting was also more prevalent in journals that endorsed reporting guidelines [35]. Recommendations to improve adherence to reporting guidelines in heath research include training on the use of reporting guidelines, improving understanding, encouraging adherence, checking adherence and providing feedback, and involvement of experts [46]. On the basis of these recommendations the instructions to authors who submit their abstracts for presentation at EOS Congress could be modified. Currently, the author guidelines recommend the submission of an abstract containing no more than 350 words using a structured format (author names and affiliations, title, aim, materials and method, results and conclusions). Under the results and conclusions sub-sections, explicit guidance could be stated requiring authors to state 95% CIs and estimates (if applicable) and to provide the clinical relevance/interpretation of their findings respectively. The effectiveness of this approach would need to be assessed by scientific panels who screen the eligibility of submitted abstracts. Furthermore, an explanation in the submission guidelines to raise awareness of reporting clinical significance in poster abstracts could be adopted. Even when CIs are reported there is a tendency to interpret them as surrogates of significance testing, or to fail to interpret them completely [47]. This implies that while journal editors and conference organizers may advocate quantification of findings over the default use of significance testing, a limited understanding of CIs and their importance in biomedical research could result in CIs being reported mechanically without meaningful interpretation. This issue could be circumvented by discussion of the study results with a statistician. Indeed, the involvement of a statistician or a methodologist is reported to increase the prevalence of confidence interval reporting in articles [30].

Duplicate data extraction has been found to be associated with reduced error rates in data collection [48]. In accordance with this and to minimize abstract misclassification and enhance accuracy, duplicate and independent data extraction was undertaken by two assessors with discussion with a third assessor in the event of any disagreements. Furthermore prior to data extraction, to reduce bias, a pilot assessment was undertaken to ensure consistency in the interpretation of the data variables that were collected. A potential limitation of this investigation is that we reviewed study poster abstracts without the assessment of published full texts of each study, therefore over-estimating the issue. Additional CIs and *P*-values could have been included in the study analyses but not reported in the conference abstracts. This issue is exemplified in a review of veterinary medicine articles in which *P*-values and CIs were only reported in 15% and 13% of abstract sections as opposed to 56% and 44% in the results section of full-text articles, respectively [31]. The current study was also limited to one congress and hence a degree of selection bias may be present as other orthodontic congresses were not assessed. A total of almost three thousand and seven hundred abstracts were assessed across a period of 10 years which we feel provides a sufficient sample to report a baseline indication of the issue and its practice over time. However, the issue of a lack of reporting of clinical significance appears to be also evident in medical conferences. In a review of RCT abstracts presented at the American Society of Clinical Oncology conference, only 26% abstracts reported the size of the treatment effect and its statistical significance [49].

We included all types of abstract study types presented at the EOS Congress so a broad assessment of the frequency of reporting clinical or statistical significance scientific poster abstracts at the EOS Congress and conclusion interpretations (based on clinical or statistical significance) could be undertaken. It could be argued that inferential statistics may not be required in particular study types such as case reports or series, narrative reviews, product reviews, and other study types such as audits. However, it should be considered that these abstract types only contributed to 12.7% of the total number of abstracts analysed in this investigation (Table 1), therefore we feel their inclusion will not significantly alter the results. We chose to report the frequency of reporting clinical or statistical significance, over a 10-year period in scientific poster abstracts presented at the EOS Congress and to assess whether the reported conclusions were drawn based on either clinical or statistical significance. Unlike, previous investigations which have reported an association between higher reporting of confidence interval reporting in articles and the involvement of a statistician [30], we could not undertake a similar analysis as the contributions of authors in presented abstracts are not commonly described.

## Conclusions

Clinical significance is often under-reported in abstracts presented at EOS and author conclusions tend to be drawn based on primarily *P*-values. While *P*-values are important in determining if a result is statistically significant, its clinical relevance is limited. The reporting of clinical significance (effect size with CI) and their interpretation in poster abstracts should be stipulated as this allows clinicians to gauge both the size and range of the observed differences between groups and the relevance to their clinical practice.

## Supplementary Material

cjaf068_Supplementary_Data

## Data Availability

The data underlying this article are available in the article, on request and in its online material.
