# Supplementary material for: Conclusions reported in European Orthodontic Congress poster abstracts: are they based on clinical or statistical significance?
Source: Eur J Orthod. 2025 Oct 22;47(6):cjaf068. doi: 10.1093/ejo/cjaf068 (PMC12540019; doi:10.1093/ejo/cjaf068)
Supplement: cjaf068_Supplementary_Data [file cjaf068_supplementary_data.zip › Supplementary FigureIV.pdf]

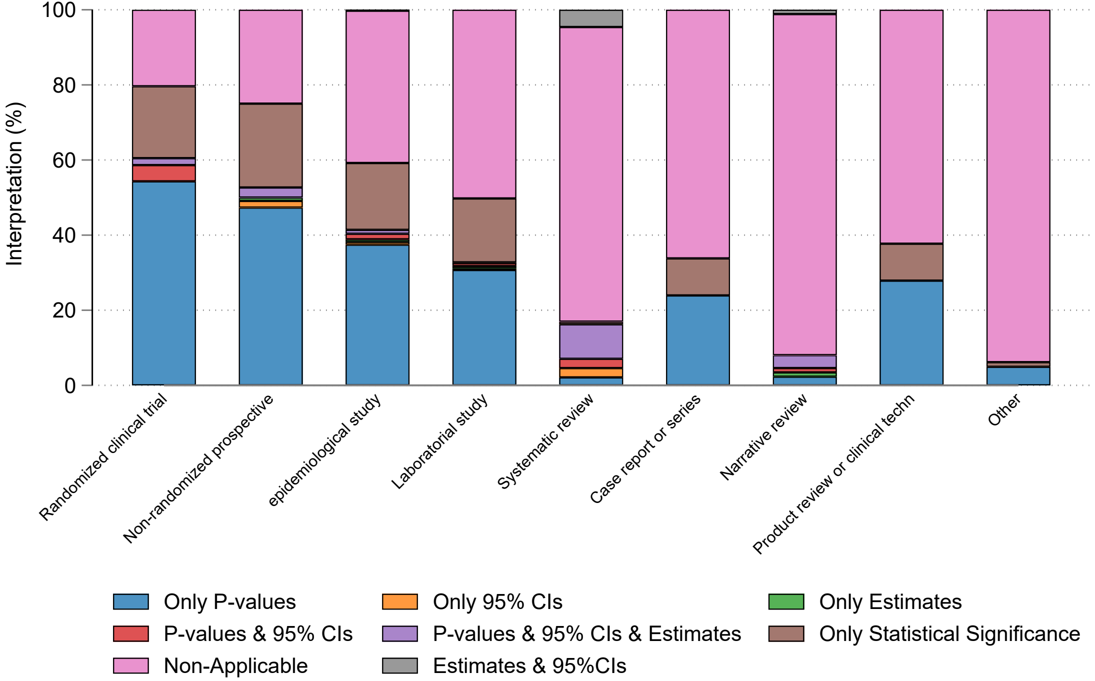

Interpretation (%)
100
80
60
40
20
0
Randomized clinical trial
Non-randomized prospective
epidemiological study
Laboratorial study
Systematic review
Case report or series
Narrative review
Product review or clinical techn
Other
Only P-values
Only 95% CIs
Only Estimates
P-values & 95% CIs
P-values & 95% CIs & Estimates
Only Statistical Significance
Non-Applicable
Estimates & 95%CIs
