# Supplementary material for: Conclusions reported in European Orthodontic Congress poster abstracts: are they based on clinical or statistical significance?
Source: Eur J Orthod. 2025 Oct 22;47(6):cjaf068. doi: 10.1093/ejo/cjaf068 (PMC12540019; doi:10.1093/ejo/cjaf068)
Supplement: cjaf068_Supplementary_Data [file cjaf068_supplementary_data.zip › Supplementary File I.docx]

Example of results and conclusions based on P-Values

INFLUENCE OF SOCIODEMOGRAPHIC AND PSYCHOLOGICAL FACTORS IN THE REPORTING OF PAIN IN A

TEMPOROMANDIBULAR JOINT DYSFUNCTION POPULATION

AIM: Pain has been defined by the International Academy for the Study of Pain as ‘A sensorial and emotional experience associated with a real or potential hurt’. Pain threshold is subjective, and sociodemographic or psychological factors play an important part in pain modulation. The aim of this study was to analyze the relevance of some sociodemographic and psychological factors in the reporting of pain in a temporomandibular joint dysfunction (TMD) population.

SUBJECTS AND METHOD: One thousand two hundred and twenty patients who reported pain in the orofacial region, sounds or limitation in mandibular movements; were explored using the Research Diagnostic Criteria for Temporomandibular Disorders (RDC/TMD). Inclusion and exclusion criteria were those include in the RDC/TMD consorce. The RDC/TMD questionnaires were used, taking as the dependent variable the ‘intensity of pain’,

obtained from: the pain of the patient at that moment, the worst pain in the last six months, and the mean pain of the last six months. All questions were measured on a visual analogue scale (VAS). A bivariate analysis was then made, analyzing its grade of association with some sociodemographic and psychological features, such as, gender, socioeconomic status, marital status, academic level, depression, anxiety or somatization. All statistic dates were obtained using the program, SAS Inc., version 9.3.

RESULTS: The main pain intensity was (5.36). With respect to the age and gender, a positive association between both factors and the reporting of pain was found, being more prevalent in females (P < 0.001) between 41 and 60 years of age (P = 0.017). A positive association between academic level and higher pain intensity was also observed (P = 0.001), with higher levels of pain in patients with lower academic abilities. Similar results

were found respect to marital status (P = 0.003), with a higher mean of pain in those who were married. No association was observed between salary level (P = 0.262) and the reporting of pain.

CONCLUSION: There is a positive association between higher reporting of pain, and being a married female, between 41 and 60 years of age, with a low academic level.

Example of results and conclusions-based P-Values, 95% CI and estinates

172 CLINICAL EFFECTIVENESS OF SURGICAL AND NON-SURGICAL APPROACHES FOR ACCELERATED ORTHODONTIC TOOTH MOVEMENT: A SYSTEMATIC REVIEW AND META-ANALYSIS

AIM: To assess the clinical effectiveness of surgical and non-surgical approaches for accelerated orthodontic tooth movement.

MATERIALS AND METHOD: Randomized controlled trials (RCTs), and controlled clinical trials (CCTs) were eligible for inclusion. Electronic search for published and unpublished studies in English, German, French or Italian was conducted in Medline, Embase, Google scholar beta, and all Cochrane Databases, at the end of November 2013 with no time restrictions. Orthognathic surgery and distraction osteogenesis interventions were excluded. Risk of bias was assessed using the Cochrane risk of bias tool for RCTs, and a specifically designed tool for CCTs. Studies with low and unclear risk of bias were mathematically combined using the random effects model.

RESULTS: Thirteen trials involving 274 participants were included. Seven trials reported on low-intensity laser (4 split-mouth RCTs, 3 CCTs), one CCT on photobiomodulation, one split-mouth RCT on pulsed electromagnetic fields, and four RCTs on corticotomy (1 parallel; 3 split-mouth). Most studies evaluated only a part of the treatment (11 assessed single tooth retraction in extraction space and 1 alignment). One trial with unclear risk of bias concluded that over the entire treatment duration low-intensity laser was more effective compared with

the conventional method (md = –167 days; 95% CI: –215.8, –118.2); average treatment duration for the control was 18.8 months (SD: 4.3). Meta-analysis of two trials indicated, during the first month of therapy, higher canine retraction rate by 0.73 mm/month with corticotomy versus the control (wmd = 0.73; 95% CI: 0.28, 1.19, P= 0.002; I2 = 46.9%, P = 0.17). Meta-analysis of three studies with an evaluation period of more than 3 months

showed a higher canine retraction rate with low-intensity laser versus the control (wmd = 0.62 mm/month; 95% CI: 0.16, 1.08, P < 0.001; predictive interval: –5.33, 6.56; I2 = 98.8%, P < 0.001).

CONCLUSION: There is some evidence that corticotomy and low laser therapy are effective, whereas the evidence for photobiomodulation or pulsed electromagnetic fields is weaker. Overall, the results should be interpreted with caution given the small number, quality, and heterogeneity of the included trials. Further research is required in this field with additional attention to adverse events and cost-benefit analysis.

280 UNIVERSITY VERSUS PRIVATE PRACTICE TREATMENT OUTCOMES EVALUATED WITH THE AMERICAN BOARD OF ORTHODONTICS OBJECTIVE GRADING SYSTEM

AIMS: To compare treatment outcomes in university versus private practice of Class I patients, using the American Board of Orthodontics Objective Grading System (ABO-OGS), and to determine whether the treatment provider choice was a significant predictor of success for the ABO examination

SUBJECTS AND METHOD: A sample of 580 Class I patients treated with or without extractions of four first premolars was subjected to discriminant analysis in order to identify a borderline spectrum of 66 patients regarding the extraction modality. Of these patients, 34 were treated in private orthodontic practice and 32 in a university graduate orthodontic clinic. The treatment outcomes were evaluated using the eight variables of the ABO-OGS.

RESULTS: The total score ranged from 10 to 47 (mean, 25.44; SD, 9.8) for the university group and from 14 to 45 (mean, 25.94; SD, 7.7) for the private practice group. The university group achieved better scores for the variables of buccolingual inclination (*P* < 0.05) and marginal ridges (*P* < 0.05) and the private practice group achieved a better score in the variable of root angulation (*P* < 0.05). However, no statistically intergroup differences were found between the total ABO-OGS scores. Additionally, no significant difference was found between the two treatment groups regarding the success rate of the ABO examination (odds ratio 0.53; 95% CI, 0.032, 8.93; *P* = 0.66).

CONCLUSION: In private practice orthodontists are more successful in angulating the roots correctly whereas in the university clinic better torque control of the posterior segments and better marginal ridges are achieved. In this sample, 71.9 per cent of the university and 79.4 per cent of the private practice patients would successfully pass the ABO examination. The treatment provider was not a significant predictor of success to the ABO examination
