# Supplementary material for: Conclusions reported in European Orthodontic Congress poster abstracts: are they based on clinical or statistical significance?
Source: Eur J Orthod. 2025 Oct 22;47(6):cjaf068. doi: 10.1093/ejo/cjaf068 (PMC12540019; doi:10.1093/ejo/cjaf068)
Supplement: cjaf068_Supplementary_Data [file cjaf068_supplementary_data.zip › Supplementary_Table 1.docx]

| **Year** |  | **2014** | **2015** | **2016** | **2017** | **2018** | **2019** | **2021** | **2022** | **2023** | **2024** | **Total** |
| --- | --- | --- | --- | --- | --- | --- | --- | --- | --- | --- | --- | --- |
| Only p-values | N | 120 | 136 | 129 | 135 | 148 | 114 | 59 | 69 | 138 | 141 | 1189 |
|  | % | 28.6% | 35.9% | 31.6% | 31.1% | 31.1% | 31.2% | 26.0% | 30.1% | 38.5% | 39.3% | 32.5% |
| Only 95% CIs | N | 4 | 2 | 3 | 1 | 4 | 2 | 1 | 3 | 5 | 4 | 29 |
|  | % | 1.0% | 0.5% | 0.7% | 0.2% | 0.8% | 0.5% | 0.4% | 1.3% | 1.4% | 1.1% | 0.8% |
| Only Estimates | N | 2 | - | - | 1 | 1 | 3 | - | - | 5 | 3 | 15 |
|  | % | 0.5% | - | - | 0.2% | 0.2% | 0.8% | - | - | 1.4% | 0.8% | 0.4% |
| P-values and 95% CIs | N | 1 | 4 | 3 | 8 | 5 | 10 | 6 | 4 | 8 | 3 | 52 |
|  | % | 0.2% | 1.1% | 0.7% | 1.8% | 1.1% | 2.7% | 2.6% | 1.7% | 2.2% | 0.8% | 1.4% |
| P-values, 95% CIs and Estimates | N | 2 | 2 | 7 | 8 | 14 | 8 | 1 | 3 | 7 | 9 | 61 |
|  | % | 0.5% | 0.5% | 1.7% | 1.8% | 2.9% | 2.2% | 0.4% | 1.3% | 2.0% | 2.5% | 1.7% |
| Estimates and 95% CIs | N | 3 | - | 2 | - | 5 | 1 | 2 | 1 | 3 | 4 | 21 |
|  | % | 0.7% | - | 0.5% | - | 1.1% | 0.3% | 0.9% | 0.4% | 0.8% | 1.1% | 0.6% |
| P-values and Estimates | N | - | - | 1 | 3 | - | 1 | 2 | 2 | 4 | 3 | 16 |
|  | % | - | - | 0.2% | 0.7% | - | 0.3% | 0.9% | 0.9% | 1.1% | 0.8% | 0.4% |
| The term “statistically significant” stated | N | 22 | - | 12 | - | 25 | 17 | 21 | 11 | 16 | 11 | 135 |
|  | % | 5.3% |  | 2.9% |  | 5.3% | 4.7% | 9.3% | 4.8% | 4.5% | 3.1% | (3.7%) |
| No inferential statistics reported | N | 265 | 235 | 251 | 278 | 274 | 209 | 135 | 136 | 172 | 181 | 2136 |
|  | % | 63.2% | 62.0% | 61.7% | 64.1% | 57.5 % | 57.3 % | 59.5 % | 59.4 % | 48.1 % | 50.5 % | 58.5% |
| Total | N | 419 | 379 | 408 | 434 | 476 | 365 | 227 | 229 | 358 | 359 | 3654 |
|  | % | 100.0% | 100.0% | 100.0% | 100.0% | 100.0% | 100.0% | 100.0% | 100.0% | 100.0% | 100.0% | 100.0% |

Supplementary Table I
