# Supplementary material for: Conclusions reported in European Orthodontic Congress poster abstracts: are they based on clinical or statistical significance?
Source: Eur J Orthod. 2025 Oct 22;47(6):cjaf068. doi: 10.1093/ejo/cjaf068 (PMC12540019; doi:10.1093/ejo/cjaf068)
Supplement: cjaf068_Supplementary_Data [file cjaf068_supplementary_data.zip › Supplementary_Table 2.docx]

| **Year** |  | **2014** | **2015** | **2016** | **2017** | **2018** | **2019** | **2021** | **2022** | **2023** | **2024** | **Total** |
| --- | --- | --- | --- | --- | --- | --- | --- | --- | --- | --- | --- | --- |
|  |  |  |  |  |  |  |  |  |  |  |  |  |
| P-values only | N | 115 | 135 | 124 | 132 | 140 | 114 | 58 | 66 | 123 | 132 | 1139 |
|  | % | 27.4% | 35.6% | 30.4% | 30.4% | 29.4% | 31.2% | 25.6% | 28.8% | 34.4% | 36.8% | 31.2% |
| 95% CIs only | N | 4 | 2 | 3 | 1 | 2 | 2 | 1 | 3 | 5 | 4 | 27 |
|  | % | 1.0% | 0.5% | 0.7% | 0.2% | 0.4% | 0.5% | 0.4% | 1.3% | 1.4% | 1.1% | 0.7% |
| Estimates only | N | 1 | - | - | 3 | - | 2 | 2 | 1 | 7 | 4 | 20 |
|  | % | 0.2% | - | - | 0.7% | - | 0.5% | 0.9% | 0.4% | 2.0% | 1.1% | 0.5% |
| P-values and 95% CIs | N | - | 4 | 3 | 8 | 5 | 9 | 6 | 4 | 7 | 3 | 49 |
|  | % | - | 1.1% | 0.7% | 1.8% | 1.1% | 2.5% | 2.6% | 1.7% | 2.0% | 0.8% | 1.3% |
| P-values, 95% CIs and estimates | N | 1 | 2 | 7 | 8 | 14 | 8 | 1 | 2 | 6 | 8 | 57 |
|  | % | 0.2% | 0.5% | 1.7% | 1.8% | 2.9% | 2.2% | 0.4% | 0.9% | 1.7% | 2.2% | 1.6% |
| The term “statistically significant” only stated without consideration of outcomes between groups | N | 84 | 84 | 72 | 78 | 48 | 46 | 27 | 33 | 33 | 33 | 538 |
|  | % | 20.0% | 22.2% | 17.6% | 18.0% | 10.1% | 12.6% | 11.9% | 14.4% | 9.2% | 9.2% | 14.7% |
| Estimates and 95% CIs | N | 3 | - | 2 | - | 3 | 1 | 2 | 1 | 3 | 4 | 19 |
|  | % | 0.7% | - | 0.5% | - | 0.6% | 0.3% | 0.9% | 0.4% | 0.8% | 1.1% | 0.5% |
| The results of inferential statistics were not considered | N | 211 | 150 | 197 | 204 | 264 | 183 | 130 | 119 | 174 | 171 | 1805 |
|  | % | 50.4 | 40.2 | 48.3 | 47.0 | 55.5 | 50.2 | 57.3 | 52.0 | 48.6 | 47.6 | 49.4 |
| Total | N | 419 | 379 | 408 | 434 | 476 | 365 | 227 | 229 | 358 | 359 | 3654 |
|  | % | 100.0% | 100.0% | 100.0% | 100.0% | 100.0% | 100.0% | 100.0% | 100.0% | 100.0% | 100.0% | 100.0% |

Supplementary Table II
