# Supplementary material for: Conclusions reported in European Orthodontic Congress poster abstracts: are they based on clinical or statistical significance?
Source: Eur J Orthod. 2025 Oct 22;47(6):cjaf068. doi: 10.1093/ejo/cjaf068 (PMC12540019; doi:10.1093/ejo/cjaf068)
Supplement: cjaf068_Supplementary_Data [file cjaf068_supplementary_data.zip › Supplementary_Table 3.docx]

| **Study Type** | | | | | | | | | | | |
| --- | --- | --- | --- | --- | --- | --- | --- | --- | --- | --- | --- |
|  |  | RCT | NRP | Epi | Lab | SR | CR | NR | Product | Other | Total |
|  |  |  |  |  |  |  |  |  |  |  |  |
|  |  |  |  |  |  |  |  |  |  |  |  |
| **Only P-values** | N | 88.0 | 53.0 | 749.0 | 195.0 | 6.0 | 17.0 | 2.0 | 17.0 | 12.0 | 1139.0 |
|  | % | 54.3% | 47.3% | 37.5% | 30.7% | 2.1% | 23.9% | 2.3% | 27.9% | 4.9% | 31.2% |
|  |  |  |  |  |  |  |  |  |  |  |  |
| **Only 95% CIs** | N |  | 2.0 | 15.0 | 3.0 | 7.0 |  |  |  |  | 27.0 |
|  | % |  | 1.8% | 0.8% | 0.5% | 2.5% |  |  |  |  | 0.7% |
|  |  |  |  |  |  |  |  |  |  |  |  |
| **Only Estimates** | N |  | 1.0 | 14.0 | 4.0 |  |  | 1.0 |  |  | 20.0 |
|  | % |  | 0.9% | 0.7% | 0.6% |  |  | 1.1% |  |  | 0.5% |
|  |  |  |  |  |  |  |  |  |  |  |  |
| **P-values & 95% CIs** | N | 7.0 |  | 29.0 | 5.0 | 7.0 |  | 1.0 |  |  | 49.0 |
|  | % | 4.3% |  | 1.5% | 0.8% | 2.5% |  | 1.1% |  |  | 1.3% |
|  |  |  |  |  |  |  |  |  |  |  |  |
| **P-values & 95% CIs & Estimates** | N | 3.0 | 3.0 | 21.0 | 1.0 | 26.0 |  | 3.0 |  |  | 57.0 |
|  | % | 1.9% | 2.7% | 1.1% | 0.2% | 9.2% |  | 3.4% |  |  | 1.6% |
|  |  |  |  |  |  |  |  |  |  |  |  |
| **Only Statistical Significance** | N | 31.0 | 25.0 | 356.0 | 108.0 | 2.0 | 7.0 |  | 6.0 | 3.0 | 538.0 |
|  | % | 19.1% | 22.3% | 17.8% | 17.0% | 0.7% | 9.9% |  | 9.8% | 1.2% | 14.7% |
|  |  |  |  |  |  |  |  |  |  |  |  |
| **Non-Applicable** | N | 33.0 | 28.0 | 811.0 | 319.0 | 222.0 | 47.0 | 79.0 | 38.0 | 228.0 | 1805.0 |
|  | % | 20.4% | 25.0% | 40.6% | 50.2% | 78.4% | 66.2% | 90.8% | 62.3% | 93.8% | 49.4% |
|  |  |  |  |  |  |  |  |  |  |  |  |
| **Estimates & 95% CIs** | N |  |  | 5.0 |  | 13.0 |  | 1.0 |  |  | 19.0 |
|  | % |  |  | 0.2% |  | 4.6% |  | 1.1% |  |  | 0.5% |
|  |  |  |  |  |  |  |  |  |  |  |  |
| **Total** | N | 162.0 | 112.0 | 2000.0 | 635.0 | 283.0 | 71.0 | 87.0 | 61.0 | 243.0 | 3654.0 |
|  | % | 100.0% | 100.0% | 100.0% | 100.0% | 100.0% | 100.0% | 100.0% | 100.0% | 100.0% | 100.0% |
|  |  |  |  |  |  |  |  |  |  |  |  |

Supplementary Table III Distribution of the interpretation of results reported in the conclusions section per study type across the study time period (RCT=Randomized clinical trial, NRP=non-randomized prospective, Epi=epidemiological study (cross -sectional, case-control, cohort, survey), Lab=laboratorial study, SR=systematic reviews (qualitative and quantitative), CR=case report or series, NR=narrative review, Product=product review or clinical technique and Other=other including audit and guidelines).
