# Supplementary figures and images for: Conclusions reported in European Orthodontic Congress poster abstracts: are they based on clinical or statistical significance?
Source: Eur J Orthod. 2025 Oct 22;47(6):cjaf068. doi: 10.1093/ejo/cjaf068 (PMC12540019; doi:10.1093/ejo/cjaf068)

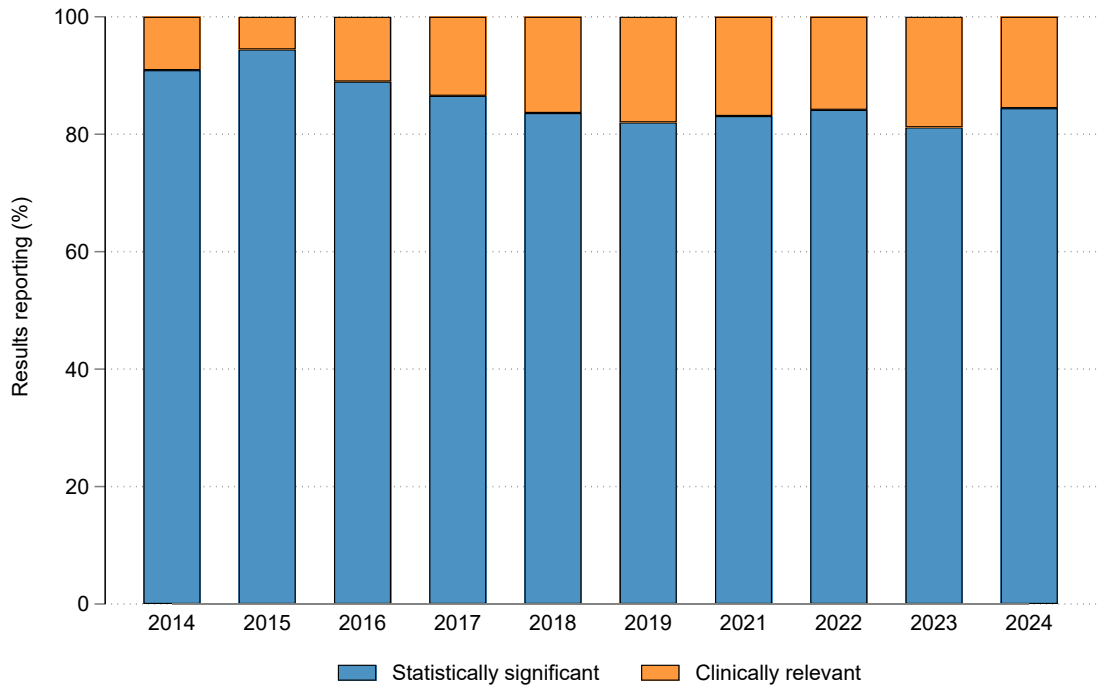

Supplement: cjaf068_Supplementary_Data [file cjaf068_supplementary_data.zip › Supplementary Figure II.pdf]

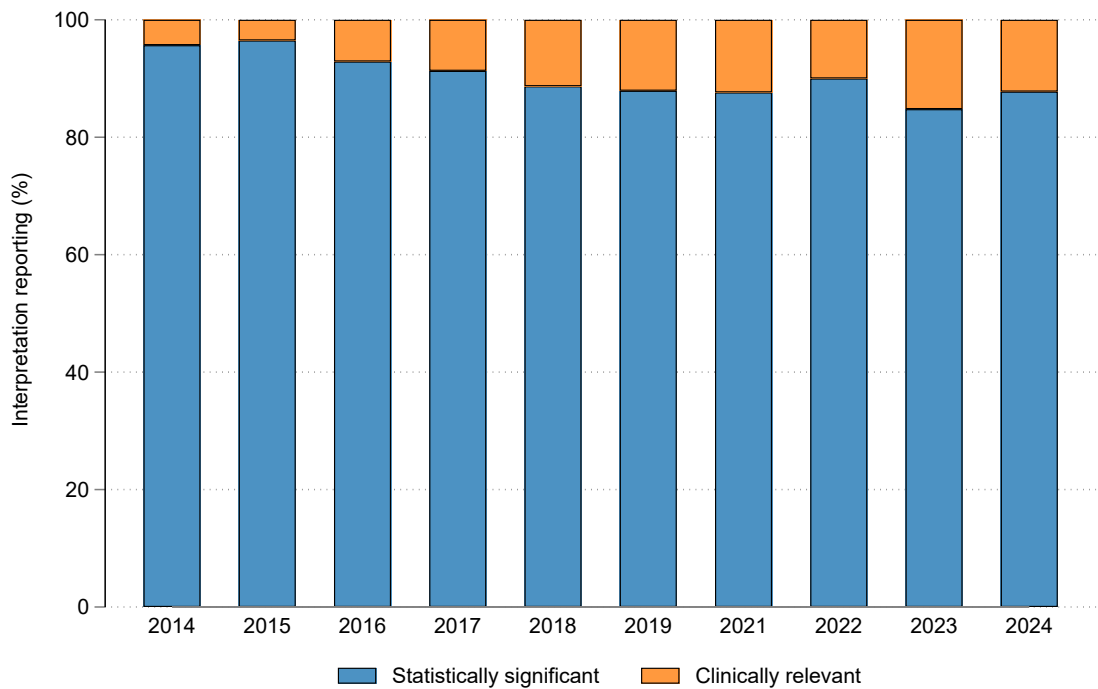

Supplement: cjaf068_Supplementary_Data [file cjaf068_supplementary_data.zip › Supplementary Figure III.pdf]

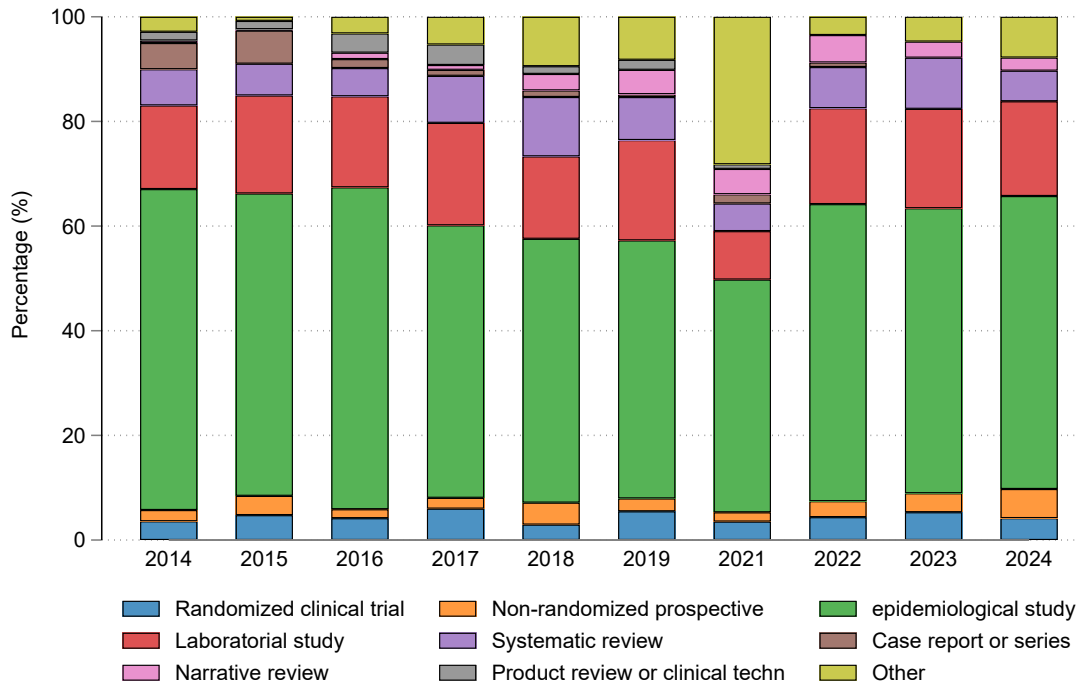

Supplement: cjaf068_Supplementary_Data [file cjaf068_supplementary_data.zip › SupplementrayFigureI.pdf]
